# Intrinsic Motivations Drive Learning of Eye Movements: An Experiment with Human Adults

**DOI:** 10.1371/journal.pone.0118705

**Published:** 2015-03-16

**Authors:** Daniele Caligiore, Magda Mustile, Daniele Cipriani, Peter Redgrave, Jochen Triesch, Maria De Marsico, Gianluca Baldassarre

**Affiliations:** 1 Laboratory of Computational Embodied Neuroscience, Istituto di Scienze e Tecnologie della Cognizione, Consiglio Nazionale delle Ricerche (LOCEN-ISTC-CNR), Rome, Italy; 2 Dipartimento di Informatica, Università di Roma “Sapienza”, Rome, Italy; 3 Department of Psychology, University of Sheffield, Sheffield, United Kingdom; 4 Frankfurt Institute for Advanced Studies, Frankfurt, Germany; University of Toyama, JAPAN

## Abstract

Intrinsic motivations drive the acquisition of knowledge and skills on the basis of novel or surprising stimuli or the pleasure to learn new skills. In so doing, they are different from extrinsic motivations that are mainly linked to drives that promote survival and reproduction. Intrinsic motivations have been implicitly exploited in several psychological experiments but, due to the lack of proper paradigms, they are rarely a direct subject of investigation. This article investigates how different intrinsic motivation mechanisms can support the learning of visual skills, such as “foveate a particular object in space”, using a gaze contingency paradigm. In the experiment participants could freely foveate objects shown in a computer screen. Foveating each of two “button” pictures caused different effects: one caused the appearance of a simple image (blue rectangle) in unexpected positions, while the other evoked the appearance of an always-novel picture (objects or animals). The experiment studied how two possible intrinsic motivation mechanisms might guide learning to foveate one or the other button picture. One mechanism is based on the sudden, surprising appearance of a familiar image at unpredicted locations, and a second one is based on the content novelty of the images. The results show the comparative effectiveness of the mechanism based on image novelty, whereas they do not support the operation of the mechanism based on the surprising location of the image appearance. Interestingly, these results were also obtained with participants that, according to a post experiment questionnaire, had not understood the functions of the different buttons suggesting that novelty-based intrinsic motivation mechanisms might operate even at an unconscious level.

## Introduction

*Intrinsic motivations* are motivations that drive human activities “for their own sake” [1], for example they drive the exploration of novel or surprising stimuli or the development of a particular skill or competence. These activities contrast with the accomplishment of valuable outcomes such as food or pain-avoidance characteristic of *extrinsic motivations* [2–5]. From an adaptive perspective, intrinsic motivation systems might have evolved to drive the acquisition of crucial knowledge and skills, even in the absence of extrinsic motivations (e.g., as in playful behaviours). Such knowledge and skills could later enhance extrinsically motivated behaviours and learning processes directly satisfying survival and reproduction [6–8].

Interdisciplinary research involving psychology, neuroscience, and computational modeling have highlighted at least three main classes of intrinsic motivation *mechanisms*. The first involves competence-based intrinsic motivations, linked to improving the agent's ability (*competence*) to modify the world through *action* [9–13]. In particular, some of the mechanisms behind intrinsic motivations might involve measures of the success in accomplishing desired *goals/tasks*. Computational models have captured these mechanisms with algorithms that estimate the probability of successfully accomplishing goals. High motivation is generated when the probability is low, or, alternatively, while probability of success increases [5,13]. The second and third class of intrinsic motivations have long been considered together and linked to the properties of *stimuli* [14], and have been called *knowledge-based intrinsic motivations* to distinguish them from competence-based intrinsic motivations [15]. Recently, the differences between two classes of knowledge-based intrinsic motivations have been stressed and clarified. Specifically, a distinction has been drawn between prediction-based and novelty-based intrinsic motivations [17,18]. *Prediction-based intrinsic motivations* are those related to predictions of a possible future state [19,20]. The computational literature captures these mechanisms with models of the world that allow agents to predict the effects of own actions on the environment [16,21]. To facilitate the interpretation of results obtained in the current study, it is important to understand that prediction-based intrinsic motivations can be distinguished further into those associated with *prediction-error* and those associated with *prediction error-improvement*. For the former, motivation is generated by the magnitude of the error of the environment-model predictions [19]; while for the latter motivation is generated by the improvement of the environment model [20]. On the other hand, n*ovelty-based intrinsic motivations* are elicited by objects, or object combinations, that have not been previously experienced and hence are not in the agent's memory. Computational models capturing these processes are typically based on the detection of anomalous/unfamiliar stimuli [22].

Note that these three types of intrinsic motivation *mechanisms* can play different *functions/roles* in cognition, but can also operate synergistically. For example, knowledge-based intrinsic motivation can lead the agent to form and select goals (e.g., visual targets), while competence-based intrinsic motivation could guide the agent to learn the skills necessary to accomplish such goals [23].

Neuroscientific research is starting to uncover the neural bases of intrinsic motivation mechanisms. Relevant for prediction-based intrinsic motivations and their role in learning motor skills, Redgrave and Gurney [24] argued that the superior colliculus (SC), a midbrain region responsible for the sensorimotor transformations required to direct gaze towards unexpected salient visual events [25,26], could be involved in computing sensory prediction errors to phasic events. Specifically, unexpected visual stimuli have been shown to elicit bursts of phasic dopamine release (an important brain neuromodulator) via a direct projection from the SC to the dopamine cell bodies in substantia nigra. These sensory-evoked bursts of dopamine are thought to be the critical teaching signals that drives trial-and-error learning in the basal ganglia [27,28]. In cases where the agent is responsible for the unexpected event, the reinforcement effect of phasic dopamine is to cause the agent to repeat and refine the action that produced the unexpected stimuli ([29]; see [30] for a model). Neurons in the SC, are sensitive to luminance changes, but not to high spatial-frequency textures, colours, and shapes, which are necessary to discriminate static novel stimuli [31]. With respect to novelty-based intrinsic motivation, the brain system most likely to categorise a high-spatial frequency stimulus or combinations of stimuli as novel is the hippocampus (Hip) [32,33,34]. When Hip detects these stimuli it can activate dopaminergic signals via a pathway involving the ventral part of the basal ganglia [33].

Intrinsic motivations have been implicitly exploited in several psychological experiments, including experiments on free visual choice with adults [35–37] and with children in developmental psychology [38]. Nevertheless, it is rarely the direct subject of investigations [39–42]. In part, this is due to the lack of formal paradigms to study intrinsic motivations. Devising experiments to investigate how intrinsic motivations operate to influence behavior is not trivial as a distinctive feature of intrinsic motivation mechanisms is their assignment of a relevance to stimuli that strongly depends on previous personal experience and continuously changes in time; e.g., how the intrinsic desire to explore the visual world influences where a child directs his/her gaze, and what may be visually relevant for him/her, depends on his/her stage of development.

Free-viewing [43,44] and gaze-contingency paradigms [45] can be used for investigating intrinsic motivations. In particular, gaze-contingency paradigms are suitable to study the role of intrinsic motivations in the discovery of how actions can cause changes in the world [45,46]. Thus, participants can learn that items displayed on a screen can be changed simply by gazing at a particular area within the screen. Gaze-contingency experiments have been used in eye-movement research with adult participants [47,48] as well as with infants [49,45]. Due to its flexibility and possibility to be used with participants of all ages, the gaze-contingency paradigm was chosen as the experimental paradigm for the present study. Here, we have used it only with adult participants, but in the future we will extend its use to study how age might affect the operation of intrinsic motivations in infants.

The current study directly investigated the effect of novelty-based and surprise-based intrinsic motivation on free exploration. Specifically, the experiment involved three conditions. The first condition investigated the effects of surprise on motivating saccades. Different levels of surprise were manipulated by letting a specific gaze direction pointing to either one of two button-like pictures to cause the appearance of a simple stimulus on a computer screen respectively at unexpected random positions or at a fixed position. The expectation was that the participants would experience a stronger motivating effect when stimuli appear at a random rather than at a fixed position as there would be a greater prediction error. The second condition investigated the motivating effects of content novelty of stimuli on foveation. Different levels of novelty were manipulated by letting a specific gaze direction pointing to either one of two button-like pictures to cause the appearance of pictures which were either always new on each trial, or consisted in simple unchanging stimulus. In this condition we expected a stronger motivational effect of novel stimuli. To evaluate their relative strength a third condition was used to directly contrast the comparative motivating effects of surprise and novelty.

## Material and Methods

A total of 24 participants (19 males, 5 females; age range 20–45 years; mean age 31.54 years) took part in the current study. They gave their written informed consent to participate to the experimental procedure and to the subsequent treatment of data. Moreover, the study was approved by the ethic committees of the “Istituto di Scienze e Techologie della Cognizione, Consiglio Nazionale delle Ricerche”. The Open Gaze Tracker and Analyzer (OGTA) system developed by the authors was used to provide quantitative measurements of the participants' gaze. OGTA is a remote low cost eye tracking system based on off-the-shelf components and open source modular software designed for use in psychological experiments (see [50] for more details).

The experiment was performed in a darkened and sound-attenuated room, with the computer screen as the only source of visible light. During testing the participants sat at a desk in front of the computer screen used to display the visual stimuli. They were asked to keep as still as possible with the head approximately 60 cm from the monitor. The eye tracker camera was positioned 5 cm underneath the monitor, and recorded the reflection of an infrared light source on the cornea relative to the pupil at a frequency of 120 Hz. Stimulus presentation was controlled by the software developed for the experiment. Each session consisted of a calibration step that preceded experimental testing.

The eye-tracker allowed for moderate head movements in a volume of 2 cm x 2 cm x 2 cm (horizontal x vertical x depth) without a significant reduction in accuracy. Blink or occlusion recovery was faster than 10 msec. During the calibration process, attractive circles were presented on the screen in a nine-point calibration sequence. The (x, y) coordinates (in pixels) of the nine calibration positions on the screen having a 1280x1024 resolution were: (20, 20), (640, 20), (1260, 20), (20, 512), (640, 512), (1260, 512), (20, 1004), (640, 1004) and (1260, 1004). The calibration procedure established an accuracy of 1 degree or less of visual angle, which corresponded to about a 2x2 cm area on the screen at a viewing distance of about 60 cm.

The visual stimuli used were images of animals or objects (“novel stimuli”), or a blue rectangle (the “simple stimulus”). In addition, two red discs were presented, which served as “buttons” that could be “pressed” by the participants directing gaze to them. Animal and object pictures were taken respectively from the National Geographic Web Site (http://www.nationalgeographic.com/) and from the Amsterdam Library of Object Images (http://staff.science.uva.nl/∼aloi/). One hundred animal and object pictures were used (50 animal pictures and 50 object pictures). The horizontal width of pictures was 400 pixels and the radius of the two button discs was 150 pixels. Areas of interest for eye tracking analysis were defined to match the position and size of the red discs and of the displayed images. Data analysis was performed with MATLAB. For each condition we used the Fourth-Spread statistical method to find and remove the outliers in the data-set. This method first identifies the boundaries of each of the quartiles in the data set. Then, it measures the fourth-spread (*fs*) as the distance between the lower and upper quartiles. The upper and lower outlier boundaries were set to 1.5 times *fs* above and below the median (see [51] for more details). This procedure removed two outliers for the condition probing the surprise-based intrinsic motivation and one outlier when probing the novelty-based intrinsic motivation.

Before being tested, the participants read the following instructions:

“*We are conducting an experiment about free visual exploration through the use of a computer screen*: *what we ask you is simply to relax and enjoy the experiment.*”

The participants knew that they were taking part in a psychological experiment, consequently they could have been extrinsically motivated to achieve whatever they guessed the goal of the experiment to be. However, our experimental design always involved the choice between two options that varied along one intrinsic motivation dimension. This means that any extrinsic motivation, if present, operated on both options, thereby avoiding any systematic bias for one of the two. Moreover, the instructions to the participants avoided creating “task-like” conditions and promoted spontaneous curiosity and exploration through visual “interaction” with the screen.

### Post experiment questionnaire

After the experiment, participants were asked to answer the following questions about their understanding of the function of the two buttons:

1. Do you feel tired?

YES NO

2. Did you like the experiment?

YES NO

3. The experiment was divided into three phases. At each stage the screen showed two red circles, one on the left and one on the right. In general, what kind of effect did they have?

4. Did you notice that looking at the two circles caused another image to appear on the screen?

YES NO

5. Did looking at the two circles have different effects in the three phases of the experiment?

YES NO

6. If so, try to indicate these effects. It is OK if you remember only few things, or if you remember them only approximately:

Phase 1: circle at left

Phase 1: circle at right

Phase 2: circle at left

Phase 2: circle at right

Phase 3: circle at left

Phase 3: circle at right

Both the instructions and the questionnaire were given in Italian and those above is their English translation.

## Experimental Procedure

Throughout the experiment two identical red discs were displayed, one on each side of the screen. By directing their gaze to one of these “buttons” participants could trigger the appearance of a new picture (animal or object) or a blue rectangle, at a fixed or random location on the screen, depending on the precise experimental configuration. A visual stimulus occurred immediately after a red disc was foveated, and stayed on the screen for 2.0 sec, during which the red buttons were disabled (i.e., had no further effect if looked at again). After the image was removed, a new trial started where the participant could again trigger one of the two buttons to elicit the appearance of a new visual stimulus, until in any condition there had been 50 such trials, i.e. the buttons had been triggered 50 times.

**Fig 1:**
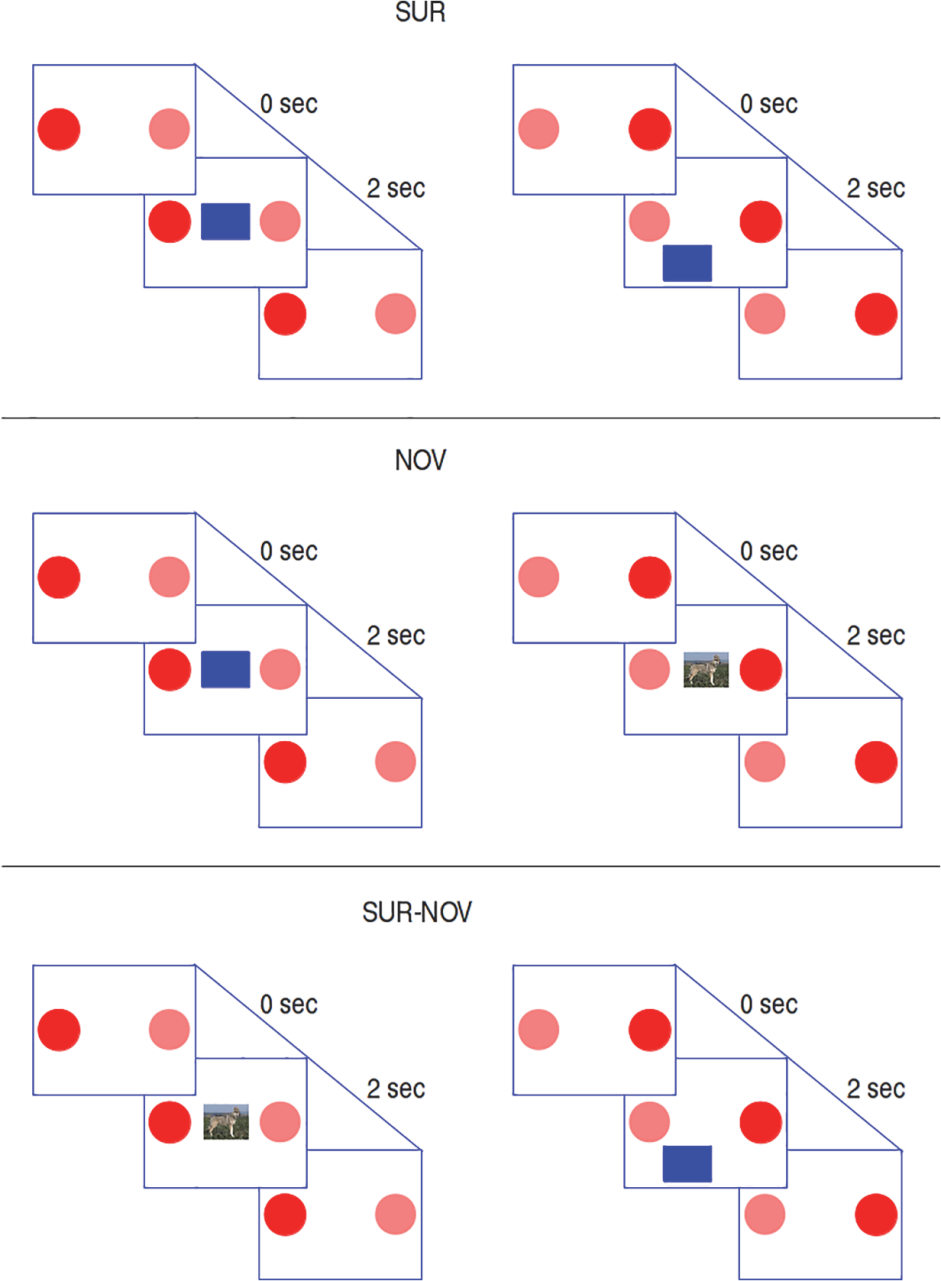
Procedure of the experiment during the three conditions. SUR condition: (left) a trial where the participant looks at the button triggering the appearance of the rectangle in the fixed position; (right) a trial where the participant looks at the button triggering the appearance of the rectangle in a random position. NOV condition: (left) a trial where the participant looks at the button triggering the appearance of the rectangle in the fixed position; (right) a trial where the participant looks at the button triggering the appearance of an always-novel picture always at the centre of the screenl. SUR-NOV condition: (left) a trial where the participant looks at the button triggering the appearance of an always-new picture always at the centre of the screen; (right) a trial where the participant looks at the button triggering the appearance of the blue rectangle that appears in random locations of the screen at each trial. For all the conditions the pictures are shown for 2.0 sec after which the buttons can be pressed again; the transparent buttons are those that the participant did not press (during the experiment the two buttons have the same appearance).

The experiment was divided into three conditions corresponding to three different consequences of gazing at the left and right buttons: (1) the first condition tested surprise-based intrinsic motivation (SUR condition); (2) the second condition tested novelty-based intrinsic motivation (NOV condition) and (3) the third condition compared the two motivations (SUR-NOV condition). The order of the conditions was balanced across participants. In the same way, the effects of the two buttons (left or right) were also balanced across participants: we applied a *Latin square* [52,53] design to have three sequences where the functions of the buttons were the same, and three mirror sequences where the functions of buttons were inverse (Tables 1, 2). Fig. 1 shows the experimental procedure including each of the three conditions.

**Table 1:** The 3 “normal” conditions of the experiment. In the “mirror” conditions the functions of the buttons were swapped.

Condition	Right Button		Left Button	
#	Position of appearance of the stimulus	Stimulus	Position of appearance of the stimulus	Stimulus
1 (SUR)	Fixed	Rectangle	Random	Rectangle
2 (NOV)	Fixed	Rectangle	Fixed	Picture (animal/object)
3 (SUR-NOV)	Fixed	Picture (animal/object)	Random	Rectangle

**Table 2:** Sequence of presentation of the stimuli during the three conditions.

Sequences	Combinations		
	A	B	C
Normal sequences (Button functions as in Table 1)	SUR/NOV/SUR-NOV 4 participants	SUR-NOV/SUR/NOV 4 participants	NOV/SUR-NOV/SUR 4 participants
Mirror sequences (Button functions swapped with respect to Table 1)	SUR/NOV/SUR-NOV 4 participants	SUR-NOV/SUR/NOV 4 participants	NOV/SUR-NOV/SUR 4 participants

### SUR condition

Looking at one of the buttons caused a simple picture (always the same—a blue rectangle) to suddenly appear at a position equidistant between the two red buttons: this position was always the same in different trials. Looking at the other button caused the same blue rectangle to appear but at different random locations on the screen in different trials. Our prediction for this condition was that the ‘random location’ button would be more attractive for the participants because the action effect would be unpredictable and therefore violate their expectations. This condition should act mainly through the prediction-based intrinsic motivation mechanism. The putative brain mechanisms responsible would likely involve the retinotopic representation of luminance changes in the superior colliculus triggering phasic dopamine responses that, because the stimuli were presented at random locations, could not be predicted.

### NOV condition

In this condition the direction of gaze to the two buttons caused the appearance of pictures always in the same place between the two red buttons. However, the fixation of one of the two buttons always caused the appearance of the same blue rectangle, while the other button caused animal/object pictures that changed from trial to trial. In this condition our prediction was that the button causing the appearance of different pictures would be more attractive as it would provide novel content information. The brain mechanisms that might underlie this process would be the capacity of hippocampus to detect complex novel patterns.

### SUR-NOV condition

In this condition one of the buttons triggered the appearance of the blue rectangle at random locations, while looking at the other button caused the appearance of a changing animal/object image at a fixed location. This condition aimed to reveal which of the two surprise/novelty motivation mechanisms is stronger.

### Rationale for the type of stimuli used

The two types of stimuli used in the experiment stress the operation of one intrinsic motivation mechanism but do not completely eliminate the operation of the other. In general, the experimental procedure was designed, (a) to balance the effect of one of the two mechanisms for the two buttons when not relevant (condition 1 and 2); (b) to test the relative strength of one system versus the other (condition 3).

Thus, in condition 1, which probed prediction-based intrinsic motivation, the presence of a novelty detection system would be balanced across the two buttons as same blue rectangle was elicited by both. Similarly, in condition 2, which probed the novelty-based intrinsic motivation, the location at which the novel and familiar images were presented remained constant. Finally, in condition 3, which contrasted the surprise- vs. novelty-based detection systems, while the two buttons may engage both systems, each would produce effects that would activate one of the systems more strongly. For example, novelty detection would be triggered by the first appearance of the blue rectangle but would rapidly habituate given the comparative simplicity of the picture. However, the surprise detection mechanism would be expected to remain highly engaged by pictures continually appearing in random locations. Alternatively, when an animal/object picture first appears it should engage the surprise detection system. But again, as the new pictures always appear at the same location this element of surprise should habituate rapidly. The novelty detection systems should, however, be continually stimulated as successive pictures were always novel.

The blue rectangle and the picture stimuli were chosen in order to have two sets of stimuli containing high and low degrees of novelty, respectively. In general, images contain information that increases with the entropy of each pixel across the different images (i.e., how much each pixel varies from image to image) and with the independence between pixels (i.e., how much the values of each pixel is uncorrelated with the values of each other pixel across the different images [50]). From this perspective, pictures made of random pixels, (i.e. with pixels each having values randomly chosen from a uniform distribution), would contain a maximum amount of information. On the other hand, such pictures would be impossible to remember because the lack of correlation between pixels would preclude patterns and regularities that the brain expects to find in natural images (e.g. edges, angles, textures, and higher-level features). Thus, for these reasons, we chose the single blue rectangle to minimise the amount of information of the displayed image, and hence its novelty, while at the same time making it easy to remember. In this case the entropy of each pixel *across images* was zero, and so each pixel value was perfectly predictable after the first experience. Moreover, the entropy of pixels *within each image* is also zero, having them always the same value and so each pixel perfectly predicts all others. This enables the participants to remember perfectly the blue rectangle after the first experience. This means that the novelty of this stimulus (intended as the difference between the stimulus memory and current stimulus perception, [18]) is minimal from the second experience onward. The use of the always-familiar blue rectangle therefore allows the novelty-based motivational effect of the always-new pictures to be very high. The blue rectangle was also chosen to probe the prediction-based intrinsic motivation system in the SUR and in the SUR-NOV while having a minimal interference from the novelty system.

### Statistical analysis

At the end of the experiment the relative frequencies of gaze shifts to the two buttons was evaluated using the t-test and a one-way analysis of variance (ANOVA). In particular, for each condition (SUR, NOV, SUR-NOV), we performed a t-test to rule out the null hypothesis for which data representing the frequency of gazing at a button were a random sample from a normal distribution with mean 25 (25 is half of the number of trials, out of 50 total trials per condition, that the button could be gazed) against the alternative possibility that the mean was not 25. The frequencies of gaze at the two buttons were fully dependent because the number of total trials was fixed (50), so it was necessary to perform the t-test on the data related to only one button to avoid an overestimation of the statistical significance of the results. The one-way ANOVA was performed to check if there was a significant difference in the gazing behavior of participants that comprehended the experiment versus those that did not comprehend it. In both type of tests a result was considered significant if the p value was less than 0.05. The comprehender/non-comprehender groups were determined on the basis of the post experiment questionnaire. A participant was classified a “comprehender” if he/she understood that looking at one of the buttons triggered the appearance of a new picture (animal or object) or a blue rectangle, in a fixed or a random position.

## Results and Discussion

### SUR condition

**Fig 2:**
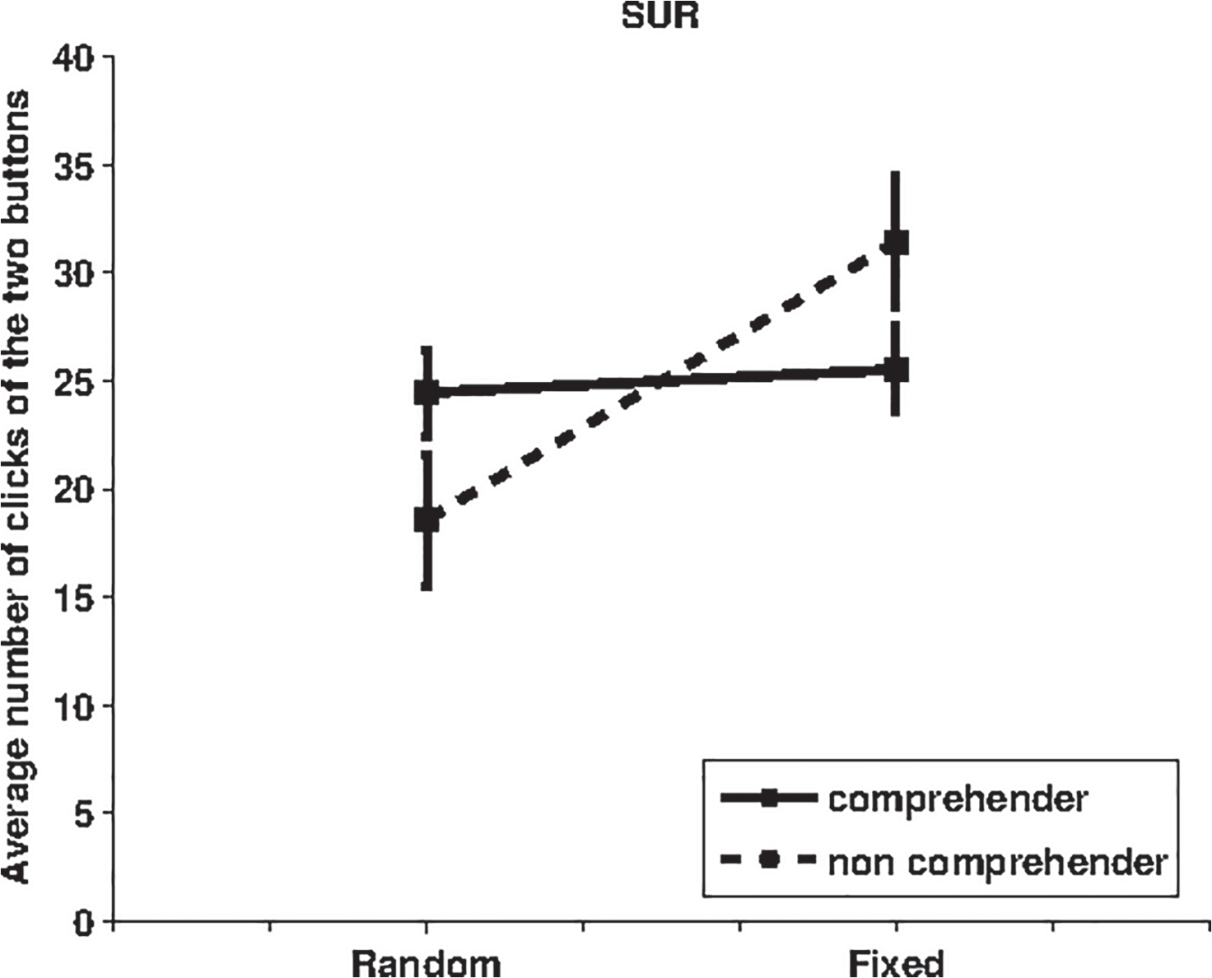
Average number of “clicks” of the two buttons for the SUR-condition. The label “Random” on the x-axis indicates the number of clicks (and standard error) of the button causing the appearance of the rectangle in a random position whereas the label “Fixed” refers to the clicks of the button which triggers the appearance rectangle in a fixed position. Data for 13 comprehenders (2 comprehenders outliers) are indicated with a solid line whereas data for 9 non-comprehenders are indicated with a dashed line.

In this condition looking at one button caused the appearance of a blue rectangle in a random position in the screen, while looking at the other button caused the appearance of a blue rectangle always at a fixed position between the two buttons. Contrary to our expectation, data illustrated in Fig. 2 show that participants had a tendency to look at the button triggering the appearance of the stimulus at the fixed position rather than at the random positions. In particular, the t-test shows that these data are close to be significant for non-comprehenders (p = 0.067, df = 8, random rectangle MEAN = 18.56, SD = 9.235; fixed rectangle MEAN = 31.44, SD = 9.235), whereas they are not significant for comprehenders (p = 0.793, df = 12, random rectangle MEAN = 24.46, SD = 7.230; fixed rectangle MEAN = 25.54, SD = 7.230). The one-way ANOVA shows that there is no significant difference between comprehenders vs. non comprehenders (p = 0.108, df = 1, F = 2.833).

This result contradicts our prediction on the presence of a surprising feature of the stimulus based on its unpredictable presentation location sufficient to activate an intrinsic motivation mechanism based on *prediction error*. Indeed, the results show how the blue rectangle appearing in the fixed rather than the random position tends to motivate gaze to be directed at its related button. A possible explanation of this result is that the fixed location of the stimulus allowed participants to *learn to predict* where it would appear. This suggests that participants may be motivated more by the *improvement of the prediction error* rather than by the *prediction error* [18]. This possibility is in line with recent studies on infants' allocation of visual resources and learning linguistic patterns. These studies have shown that with seven/eight-month-old infants the probability of looking away is greater for stimuli sequences whose complexity is either very low or very high [40]. Similarly, it has been demonstrated that 17-month-old infants attend longer to learnable, compared to unlearnable linguistic patterns [41]. While these studies do not explicitly refer to intrinsic motivations, they interpret the results on the basis of an *inner mechanism* that could drive the behaviour of infants to seek proper levels of experience novelty, thus avoiding overly simple or complex events [40]. Infants might indeed be sensitive to their learning progress and preferentially direct their attention to learnable aspects of their environment [41].

Future work could further investigate the prediction error improvement mechanism possibly involved in our experiment by manipulating the predictability of the items' appearance. For example, it could contrast a condition where a stimulus appears in a fixed position with a condition where it appears in a sequence of positions having a regular pattern (e.g., a circular pattern).

### NOV condition

**Fig 3:**
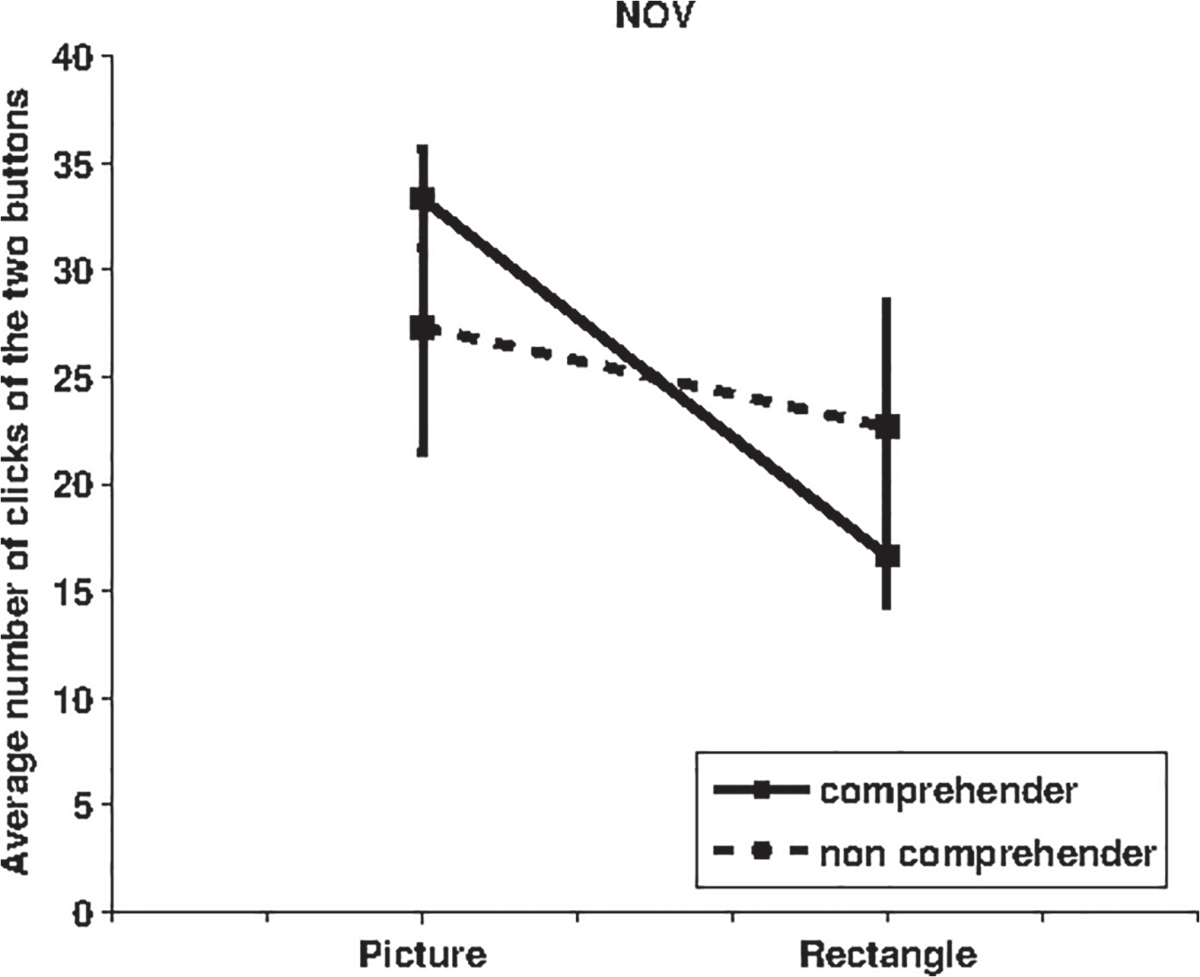
Average number of “clicks” of the two buttons for the “NOV condition”. The label “Picture” on the x-axis indicates the number of clicks (and standard error) for the button causing the appearance of the picture changing at each trial, whereas the label “Rectangle” refers to the data for the button which triggered the appearance of the rectangle. Data for 14 comprehenders (1 outlier) are indicated with a solid line whereas data for 9 non-comprehenders are indicated with a dashed line.”

In this condition, the contrast caused by gazing at the two buttons was between the appearance of same blue rectangle and different pictures (e.g., animals or objects), both always appearing in the same fixed position. Our expectation was that the pressure of the button causing the appearance of novel pictures would have been more motivated. The data shown in Fig. 3 support this prediction. The t-test shows a significant effect for comprehenders (p = 0.003, df = 13, fixed rectangle MEAN = 16.64, SD = 8.661; fixed picture MEAN = 33.36, SD = 8.661), whereas the result for non-comprehenders is not statistically significant (p = 0.7, df = 8, fixed rectangle MEAN = 22.67; SD = 17.53, fixed picture MEAN = 27.33, SD = 17.53). The one-way ANOVA shows that there is no significant difference between comprehenders and non-comprehenders (p = 0.283, df = 1, F = 1.216).

The results in Fig. 3 confirm the prediction that the button causing the appearance of different pictures was more attractive for the participants, although this did not hold for the non-comprehenders. The result for the comprehenders should depend on the novelty of the pictures that furnishes new information to the participants, e.g. allows them to store in memory novel interesting patterns. The negative result for the non-comprehenders might instead depend on the fact that although the novel pictures might be more interesting, these participants are not able to learn how to actively cause them.

### SUR vs NOV condition

**Fig 4:**
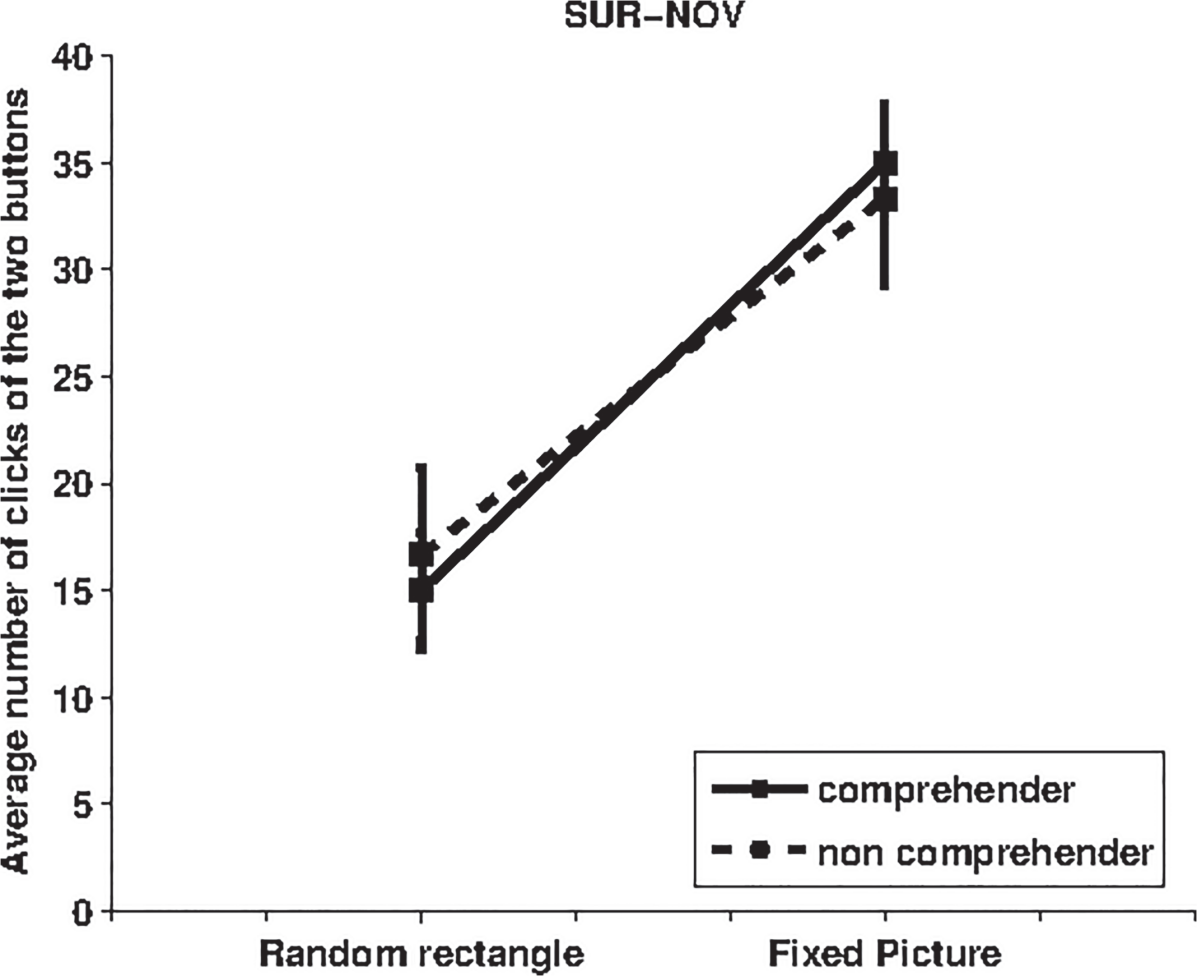
Average rate of “clicks” of the two buttons for the SUR-NOV condition. The label “Random rectangle” on the x-axes indicates the number of clicks (and standard error) for the button causing the appearance of the blue rectangle in a random position whereas the label “Fixed picture” refers to the data for the button which triggered the appearance of the picture in a fixed position. Data for 15 comprehenders are indicated with a solid line whereas data for 9 non-comprehenders are indicated with a dashed line.

In this condition the comparison was between the appearances of the simple picture (blue rectangle) in different locations with different complex pictures appearing in a fixed position. This condition aimed to contrast the intrinsic motivation mechanisms based on surprise and novelty. Given the result involving surprise was negative, the current condition confirmed the stronger influence of the intrinsic motivation based on novelty. Fig. 4 shows that participants strongly preferred gazing at the button that caused the appearance of the complex pictures. The t-test shows a significant effect for comprehenders (p = 0.003, df = 14, random rectangle MEAN = 15, SD = 10.64; fixed picture MEAN = 35, SD = 10.64) and a strong trend for non-comprehenders (p = 0.074, df = 8, random rectangle MEAN = 16.67, SD = 12.19, fixed picture MEAN = 33.33, SD = 12.19). The one-way ANOVA shows no statistically significant difference between comprehenders and non comprehenders (p = 0.728, df = 1, F = 1.124).

The results shown in Fig. 4 confirm that the intrinsic motivation mechanism sensitive to the novelty of image content is stronger than the appearance of familiar images at surprising locations. Interestingly, in the SUR-NOV condition the similarity between the data obtained from participants that have not understood the functions of the two buttons and participants that have understood such functions suggest that novelty-based intrinsic motivation mechanisms could be operating at an unconscious level. This outcome, in line with that of the novelty alone, deserves further investigation.

## Conclusion and Future Research

As far a we know, this is the first empirical study to directly investigate the interplay between two intrinsic motivation mechanisms, one based on a familiar stimuli appearing at surprising locations and the other based on novel stimuli appearing at a familiar location. The contrast between these two conditions was in their ability to intrinsically motivate the acquisition and maintenance of gaze behavior directed to two visual targets. The demonstrated predominance of novelty-based intrinsic motivation suggests a possible involvement of the hippocampus [33]. The comparative ineffectiveness of prediction-error-based (or location-based surprise) intrinsic motivation suggests, in the current conditions, a comparatively weak input from retinotopic visual structures (e.g. the superior colliculus) to the brain’s action selection and reinforcement learning systems. In this respect, the results obtained in the SUR condition suggest that prediction-improvement rather than simple prediction error magnitude could be more intrinsically motivating. In the current experimental conditions, mere surprise seemed thus insufficient to trigger intrinsic motivation based on prediction error. Perhaps this system is better designed to drive the learning of regular patterns of observed events, rather than a series of random locations that cannot be learnt. In contrast, the regular appearance of the blue rectangle in the fixed position could have facilitated the appreciation of the contingency between the gaze and stimulus appearance. Further research will be needed to elucidate this possibility further.

The work also highlights the importance of entertaining the possibility that intrinsic motivation mechanisms involving stimulus features including novelty/familiarity/surprise might operate at an unconscious level. This is a second issue that deserves further investigation.

Finally, the experiment shows that the gaze contingency paradigm can be used effectively to investigate the influence of intrinsic motivations on the acquisition of visual skills. Moreover, the current study provides a sound base from which the same processes can be investigated in babies, with a view to identifying a developmental profile of how intrinsic motivations can drive learning and behavior in different age groups.
